# CAF-derived exosome-miR-3124-5p promotes malignant biological processes in NSCLC via the TOLLIP/TLR4-MyD88-NF-κB pathway

**DOI:** 10.32604/or.2024.054141

**Published:** 2024-12-20

**Authors:** TAO SUN, QINGHUA SONG, HUA LIU

**Affiliations:** 1 Department of Respiratory and Critical Care Medicine, Affiliated Hospital of Nantong University, Medical School of Nantong University, Nantong, 226001, China; 2 Department of Emergency, The People’s Hospital of Rugao, Nantong, 226500, China; 3 Department of Haematology, The People’s Hospital of Rugao, Nantong, 226500, China

**Keywords:** Non-small cell lung cancer (NSCLC), Cancer-associated fibroblasts (CAFs), Exosomes, miR-3124-5p, Toll-interacting protein (TOLLIP)

## Abstract

**Background:**

Lung cancer is a life-threatening disease that occurs worldwide, but is especially common in China. The crucial role of the tumour microenvironment (TME) in non-small cell lung cancer (NSCLC) has attracted recent attention. Cancer-associated fibroblasts (CAFs) are the main factors that contribute to the TME function, and CAF exosomes are closely linked to NSCLC.

**Methods:**

The expression levels of miR-3124-5p and Toll-interacting protein (TOLLIP) were analysed by bioinformatics prediction combined with RT-qPCR/Western Blot detection. Fibroblasts were isolated and identified from clinical NSCLC tissues. Transmission electron microscopy and Western Blot were used to identify exosomes from these cells. Changes in proliferation (CCK-8 and clone formation), migration (wound healing), and invasion (transwell) of NSCLC cells were measured. The Luciferase reporter test was applied to clarify the binding of miR-3124-5p to TOLLIP. The TOLLIP/TLR4/MyD88/NF-κB pathway proteins were determined using Western blot analysis.

**Results:**

MiR-3124-5p is overexpressed in clinical tissues and cells of NSCLC. MiR-3124-5p was dramatically enriched in CAF-derived exosomes. Cellular experiments revealed that CAFs delivered miR-3124-5p into NSCLC cells via exosomes, stimulating cancer cell progression. MiR-3124-5p acted as a sponge to negatively regulate TOLLIP expression, which activated the TLR4/MyD88/NF-κB axis to promote the occurrence and development of NSCLC. Functional salvage tests were performed to determine whether CAF-exosome-derived miR-3124-5p plays a pro-cancer role in NSCLC by affecting the TOLLIP signalling pathway.

**Conclusions:**

These results provide an interesting direction for the diagnosis and therapy of NSCLC.

## Introduction

Lung cancer seriously endangers the life and health of the Chinese people [1]. NSCLC is the principal subtype of lung cancer [2]. Annually, approximately 2 million new lung cancer patients are diagnosed [3], and approximately half of these new cases are in Asia [4]. The incidence rate of lung cancer has been declining in the US for nearly a decade, but it is increasing in China [5]. Despite some encouraging treatment results, the mortality rate remains high. Metastasis is the major driving force behind lung cancer-related deaths [6]. The metastatic process covers several complex processes, including cancer cell proliferation and invasion at the primary site, migration to distal organs or tissues via circulation, and epithelial-mesenchymal transition (EMT) [7]. Therefore, elucidating the molecular pathways that affect tumour cell metastasis has important clinical value for improving the prognosis of NSCLC patients.

Current research has focused on various signalling pathways in cancer cells [8]. However, the malignant processes of cancers involve tumour cells with disrupted signalling axes and a complex tumour microenvironment (TME) [9]. The TME is an important driver of cancer progression, and it comprises tumour, stromal, and immune cells, which produce and secrete numerous factors [10]. In TME, CAFs are the major enforcers, stimulating the development of tumours by producing oncogenic signals [11]. CAFs play a critical role in tumour malignant phenotype and treatment response [12]. For example, CAFs facilitate cancer growth by releasing proangiogenic factors that induce angiogenesis [13]. PD-L1-positive CAFs regulate NSCLC cell stem cell-like characteristics by delivering HGF, resulting in chemotherapy resistance [14]. In TME, more infiltration of CAFs indicates a poorer prognosis for lung cancer patients [15]. CAFs have various functions, such as reshaping the extracellular matrix (ECM), secreting cytokines, promoting EMT, and communicating signals with tumour cells, thus facilitating cancer occurrence, development, and drug resistance [16]. Metastasis accounts for a high proportion of death in patients with NSCLC, and it exceeds primary tumour mortality [17,18]. CAFs participate in the development, metastasis, and chemoresistance of tumour cells by delivering exosomes [19]. Therefore, studying the molecular mechanisms of CAFs in the TME regulation of malignant processes, like NSCLC metastasis, is highly valuable for the clinical treatment of NSCLC.

Among the substances secreted by CAFs, exosomes are a major type. Exosomes act as a bridge for signal crosstalk between TME and cancer cells [12]. Exosomes are small lipid vesicles approximately 40–120 nm in size that contain many biomolecules, such as DNA and RNA [20]. Many types of cells secrete exosomes, which are found in urine, blood, and other body fluids, to facilitate cell-to-cell interactions [21]. The biomolecules encapsulated in exosomes are relatively stable and not easily degraded, and they participate in “high-fidelity” signalling between local cell populations or distant tissues and organs [22]. With an increasing understanding of non-coding RNAs, some researchers have proposed that CAF-derived miRNAs interact with the 3′-UTR of messenger RNAs (mRNAs) to suppress the expression of target genes [23]. CAF-derived exosomal miRNAs are closely connected with the malignant processes of cancer cells [24–26]. For instance, CAF-mediated exosomal miR-106b facilitates pancreatic cancer cell chemotherapy resistance by silencing TP53INP1 [27]. Exosomal miR-210 secreted by CAFs accelerates the progression of NSCLC via the regulation of PTEN [7]. Exosomal miR-103a-3p from CAFs inhibits NSCLC apoptosis and stimulates chemoresistance [28]. These studies elucidated the function of miRNAs derived from CAF exosomes in NSCLC, but the regulatory pathways involved are not clear. Therefore, this study aims to further explore novel oncogenic miRNAs secreted by exosomes of CAFs and elucidate their regulatory mechanisms in NSCLC.

## Materials and Methods

### Bioinformatics analysis

MiRNAs with prominent differences in expression between NSCLC patients and normal controls were identified using GEO expression profile (GSE171517 and GSE135918) analysis (http://www.ncbi.nlm.nih.gov/geo, accessed on 11 June 2024). In brief, we divided the samples into cancer group and normal group according to the type of samples, and conducted online analysis through GEO2R online platform (GSE171517: https://www.ncbi.nlm.nih.gov/geo/geo2r/?acc=GSE171517 (accessed on 22 February 2023); GSE135918: https://www.ncbi.nlm.nih.gov/geo/geo2r/?acc=GSE135918 (accessed on 11 June 2024)) to screen the differentially expressed genes of *p*adj (the adjusted *p*-value) < 0.05. The miR-3124-5p in exosomes was detected using the Vesiclepedia website (http://microvesicles.org/index.html, accessed on 11 June 2024). TargetScanHuman 7.2 (https://www.targetscan.org/vert_80/, accessed on 11 June 2024) and miRWalk (http://mirwalk.umm.uni-heidelberg.de/, accessed on 11 June 2024) (chose |energy| ≥ 25) were applied to forecast targets of miR-3124-5p. UALCAN website (http://ualcan.path.uab.edu/, accessed on 11 June 2024) was applied to analyse Toll-interacting protein (TOLLIP) expression in NSCLC (including lung adenocarcinoma (LUAD) and lung squamous cell carcinoma (LUSC)). The Kaplan-Meier Plotter (http://kmplot.com/analysis/index.php?p=background, accessed on 11 June 2024) was applied to analyse the affect between TOLLIP and overall survival (OS) in NSCLC patients.

### Clinical samples

A total of 20 surgical tissue samples were gathered from patients diagnosed with NSCLC in Rugao People’s Hospital from June to December 2023. The clinicopathological information is shown in Table 1. Before the surgery, these patients did not undergo chemotherapy or radiotherapy. All patients have signed informed consent forms. These clinical tissues (cancer and corresponding paracancerous) were first frozen in liquid nitrogen and then stored at −80°C for no more than 3 months.

**Table 1:** Pathological features of NSCLC patients

	Samples (n = 20)
**Age median (range)**	
≤66	11
>66	9
**Gender**	
Male	7
Female	13
**Smoking history**	
No	5
Yes	15
**Tumour stage**	
T1	15
T2	3
Not recorded	2
**Tumour size (cm** ^**3**^ **)**	
<5	12
≥5	8
**Metastasis**	
No	15
Yes	5

### Cell lines and culture conditions

ATCC (USA) supplied the lung normal epithelial cells (BEAS-2B (cat.no. CRL-3588)) and NSCLC cells (including A549 (cat.no. CCL-185), NCI-H1299 (cat.no. CRL-5803), HCC827 (cat.no. CRL-2868) and NCI-H838 (cat.no. CRL-5844)). All cell lines were identified, and no mycoplasma contamination was detected. DMEM (Pricella, cat.no. PM150210, China) was applied to culture these cells. The 10% foetal bovine serum (FBS) (Solarbio, cat.no. S9020, China) and 1% antibiotics (Beyotime, cat.no. C0222, China) were added in the media. These cells were cultured at 37°C in 5% CO_2_ (Thermo Scientific™ BB 150 CO_2_ Incubator, USA).

### Extraction, culture and identification of CAFs

The extraction and analysis of CAFs and normal fibroblasts (NFs) were based on previous experimental methods [29]. The cancer tissue of NSCLC patients was collected to extract CAFs, and the paracancerous tissue of the same patient approximately 5 cm away from the cancer area was used to extract NFs. In brief, the collected clinical samples were cleaned with pre-cooled sterile 1 × PBS, then shredded and digested with 0.1% collagenase (Sigma, cat.no. C0130, USA) at 37°C for 3 h. After centrifugation, wash with DMEM medium and culture with DMEM medium (adding 20% FBS) for 2 d at 37°C in 5% CO_2_. The adherent cells are preserved (suspended cells are removed). Western blot and immunofluorescence (IF) were applied to evaluate biomarkers of CAFs, including vimentin (anti-vimentin (1:1000, cat.no. ab137321, Abcam, UK)), α-smooth muscle actin (α-SMA, anti-α-SMA (1:1000, cat.no. #19245, CST, USA)) and fibroblast activating protein (FAP, anti-FAP (1:1000, cat.no. ab314456, Abcam)). All primary fibroblasts were passaged three times and subsequent experiments were conducted.

### Isolation and detection of CAF-exosomes

CAF exosomes derived from NSCLC cells were extracted according to previously reported methods [7]. In brief, the exosomes of primary CAFs and NFs were extracted by ultracentrifugation (Beckman, Optima XPN-100, USA), and the supernatant was taken for continuous gradient centrifugation (2000 g × 30 min, 10,000 g × 40 min, and 100,000 g × 70 min, respectively, at 4°C). Finally, the precipitate was rinsed with 1 × PBS and centrifuged again (100,000 g × 70 min, at 4°C). After centrifugation, it was suspended with 1 × PBS and kept at −80°C. The features of the purified exosome ultramicrovesicles was examined by transmission electron microscopy (TEM, HITACHI, HT7700, Japan). The CD9 (anti-CD9 (1:1000, cat.no. ab236630, Abcam)) and TSG-101 (anti-TSG-101 (1:1000, cat.no. ab133586, Abcam)) in NFs and CAFs expression levels were tested using Western blot. These two indicators are specific markers of exosomes.

### Tracking exosomes

The exosomes were labeled (emitting green fluorescence) using a PKH67 fluorescent probe (Sigma, cat.no. MIDI67-1KT, USA), and the presence of CAF-derived exosomes in NSCLC cells was visualised. In short, the extracted exosomes were diluted and suspended with diluent, followed by incubation with PKH67 dye solution. After incubation for 5 min, serum was added to end the reaction, and 1 × PBS was used for washing. NSCLC cells were inoculated into 6-well plates at a rate of 1 × 10^5^ cells/well, and 10 μg of the labeled exosomes were added to each well for co-incubation. After incubating at room temperature for 24 h, DAPI was added to stain the nucleus. Finally, fluorescence microscope (Leica, TCS SP5, Germany) was used for observation.

### Cell transfection

The cell transfection experiments were performed according to previous experimental methods [30]. In brief, CAFs or NSCLC cells were cultured (The cells were cultured in serum-free DMEM medium for 24 h at 37°C in 5% CO_2_ and then replaced with complete medium) at appropriate concentrations (1 × 10^6^ cells/well), and the transfection reagent Lipofectamine 3000 (5 μL, Thermo Fisher, cat.no. L3000001, USA) and oligonucleotides, including the miR-3124-5p mimic and inhibitor, were came from GenePharma (Shanghai, China). The specific sequences: mimic, 5′-UUCGCGGGCGAAGGCAAAGUC-3′; mimic-NC, 5′-ACUACUGAGUGACAGUAGA-3′; inhibitor, 5′-GACTTTGCCTTCGCCCGC-3′ and inhibitor-NC, 5′-CAGUACUUUUGUGUAGUACAA-3′. A pcDNA3.1 overexpression vector containing full-length human TOLLIP (pcDNA3.1-TOLLIP) and the negative control (pcDNA3.1-NC) were obtained from GenePharma. As an NC, a pcDNA3.1 vector that was empty was utilised. The TOLLIP overexpression plasmid was used at 2 µg/mL, and the miR-3124-5p mimic/inhibitor was used at 50 nmol/L. These components were mixed together and transfected into the above cells at 37°C for 24 h. After 48 h, the cells were saved for the subsequent experiments.

### RT-qPCR experiments

RT-qPCR experiments were performed according to previous reports [31]. In brief, TRIzol reagent (Invitrogen, cat.no. 15596026CN, USA) was applied to obtain total RNAs from tissues, cells and exosomes, and Prime Script RT reagent Kit (Takara, cat.no. RR037A, Japan) was used to synthesize the first-strand cDNAs. The miRNA First-Strand cDNA Synthesis Kit (TIANGEN, cat.no. KR211, China) was applied to synthesize miRNA cDNAs. RT-qPCR was used to test the TOLLIP gene expression using SYBR Green reagent (Thermo Fisher, cat.no. A25742, USA). RT-qPCR for miR-3124-5p was coducted utilizing the miRcute Plus miRNA qPCR Detection Kit (TIANGEN, cat.no. FP411, China). The relative expression levels of these genes were tested via the 2^−ΔΔCt^ method. The results were normalised to those of U6 and GAPDH. The primer sequences of these genes were designed and synthesized by GenePharma (Table 2). Specific steps: First, pre-denature for 15 min (95°C), then react for 40 cycles (denature at 95°C for 10 s, annealing at 60°C for 20 s, and finally extend at 72°C for 20 s).

**Table 2:** Primers for RT-qPCR

Targets	Sequences	
U6	F: 5′-CTCGCTTCGGCAGCACA-3′	R: 5′-AACGCTTCACGAATTTGCGT-3′
miR-3124-5p	F: 5′-ATTCGCGGGCGAAGGC-3′	R: 5′-AGTGCAGGGTCCGAGGTATT-3′
TOLLIP	F: 5′-TGGGCCGACTGAACATCAC-3′	R: 5′-GTGGATGACCTTATTCCAGCG-3′
GAPDH	F: 5′-GAAGGTGAAGGTCGGAGTC-3′	R: 5′-GAAGATGGTGATGGGATTTC-3′

### Western blot

Total proteins were collected from NFs and CAFs, fibroblast-derived exosomes and NSCLC (including A549 and HCC827) cells using RIPA lysis buffer (Beyotime, cat.no. P0013B, China). In brief, 200 μL of pre-cooled RIPA lysis buffer was used to lyse cells or exosomes. After 30 min of lysis on ice, the lysed cells or exosome suspension were transferred to the centrifuge tubes, and an ultrasonic disruptor (Scinetz, IID, China) was used to briefly sonicate (Power of 500 W, ultrasound for 3 s, interval of 7 s, continuous for 20 cycles, all operations performed on ice) the cell lysate for additional cell lysis and enhanced protein release. These lysed cells or exosome suspensions were then centrifuged (12,000 × 10 min) to remove cell debris and insoluble impurities. Finally, the supernatants containing total proteins were carefully collected. The BCA kit (Beyotime, cat.no. P0010S, China) was used to test the protein concentrations. The Western blot experimental steps were based on previously report [30]. In brief, SDS-PAGE (Solarbio, cat.no. P1200, China) was applied to isolate the proteins and moved them to nitrocellulose membranes (Millipore, cat.no. 71078, USA). Then, milk powder was used to block the membranes and incubated with different antibodies (the first and corresponding second antibodies are added in order). An enhanced chemiluminescence (ECL) system (Thermo Fisher, cat.no. 32134, USA) was used to visualize the blots. The specific antibodies information: anti-FAP (1:1000, cat.no. ab314456, Abcam), anti-α-SMA (1:1000, cat.no. #19245, CST), anti-vimentin (1:1000, cat.no. ab137321, Abcam), anti-CD9 (1:1000, cat.no. ab236630, Abcam), anti-TOLLIP (1:1000, cat.no. ab133586, Abcam), anti-TSG-101 (1:1000, cat.no. ab133586, Abcam), anti-TLR4 (1:1000, cat.no. ab187198, Abcam), anti-MyD88 (1:1000, cat.no. ab28763, Abcam), anti-NF-κB p65 (1:1000, cat.no. ab32536, Abcam), anti-GAPDH (1:2500, cat.no. ab9485, Abcam) and HRP-conjugated secondary antibodies (1:10,000, cat.no. ab205718, Abcam).

### Cell counting kit-8 (CCK-8)

A CCK-8 kit (Solarbio, cat.no. CA1210, USA) was used to examine the proliferation of the NSCLC cells after incubation with CAF-derived exosomes and/or transfection with different oligonucleotides/plasmids. The experimental procedures were based on a previous report [30]. In brief, 2 × 10^3^ cells/well of A549 and HCC827 cells were cultured in DMEM medium (containing 10% FBS) for 0, 24, 48, and 72 h in 96-well plates at 37°C in 5% CO_2_, then treated with CCK-8 (10 μL) and the absorbance at 450 nm was observed using a microplate reader (BIO-RAD, iMark-680, USA).

### Colony formation

A549 and HCC827 cells were inoculated in a 6-well plate (Corning, cat.no. CLS3335, USA) at a concentration of 1000 cells/well and cultured at 37°C for 7 days after different treatments. The NSCLC cells were immobilized with methanol (100%, Sangon Biotech, cat.no. A506806, China) for 15 min, and then stained with crystal violet (1%, Beyotime, cat.no. C0121, USA) for 10 min. The stained colonies were photographed using A digital camera (Nikon, D810, Japan) and counted by the naked eye.

### Wound healing assay

The cell migration ability was observed through a wound healing assay [32]. In brief, the NSCLC cells were inoculated in a 6-well plate (5 × 10^5^ cells/well), and a linear scratch was created using a 10 μL pipette tip. 1 × PBS was used to remove the detached cells. 2% FBS was added to the medium for subsequent culture. A microscope (IX71, Olympus, Japan) was used to capture images (40×) at 0 and 24 h for the evaluation of the cell migration ability.

### Transwell assay

The cell invasion ability was observed through a Transwell assay [32]. In brief, Matrigel (BD Biosciences, cat.no. 354324, USA) was coated on a Transwell chamber (8 μm, Corning, cat.no. CLS3422) and then it was placed in a 24-well plate. The upper chamber contained an appropriate concentration (2 × 10^4^ cells/well) that was suspended and inoculated using serum-free medium. In the lower chamber, the conditioned medium was added. After culture 48 h, the cells were immobilized with paraformaldehyde (4%, Beyotime, cat.no. P0099, China), and then stained with 1% crystal violet for 10 min. The inverted microscope (IX71, Olympus, Japan) was used to capture the images, and the NSCLC cell invasion ability was calculated. Under the microscope, observe and count 5 randomly selected areas on the membrane surface of the Transwell chamber to calculate the invasion ability of cells.

### Luciferase reporter assay

The psiCHECK-2-TOLLIP 3′-UTR-wild-type (WT) and -mutant (MUT) luciferase reporter vectors were designed and constructed from GenePharma. Site sequences: TOLLIP (WT): CCUUCGCCCGC, TOLLIP (MUT): GGAAGCGGGCG. First, HEK293T (ATCC, cat.no. CRL-3216) were seeded into 24-well plates and after 24 h incubation the confluence reaches to 60%–70%. Then, the HEK293T cells were co-transfected with 20 nM miR-3124-5p mimic or mimic-NC together with 0.1 μg psiCHECK-2-TOLLIP-WT or psiCHECK-2-TOLLIP-MUT by Lipofectamine 3000. After 48 h, the dual luciferase reporter gene assay was used to determine the luciferase activity (Promega Corporation, cat.no. E1910, USA) and standardized to Renella luciferase activity.

### Statistical analysis

The mean ± standard deviation (SD) calculated from all experimental data (three replicates) was statistically analysed using GraphPad Prism (v9.0.0.121, CA, USA). Student *t*-test was employed to calculate the statistical differences between the two groups. ANOVA was applied for multiple groups. The Kaplan-Meier was applied to analyse survival. The log-rank test was used to analyse the differences between the TOLLIP high-expression and low-expression groups. *p* < 0.05 was considered to indicate a statistically significant result.

## Results

### MiR-3124-5p was overexpressed in lung cancer

**Figure 1:**
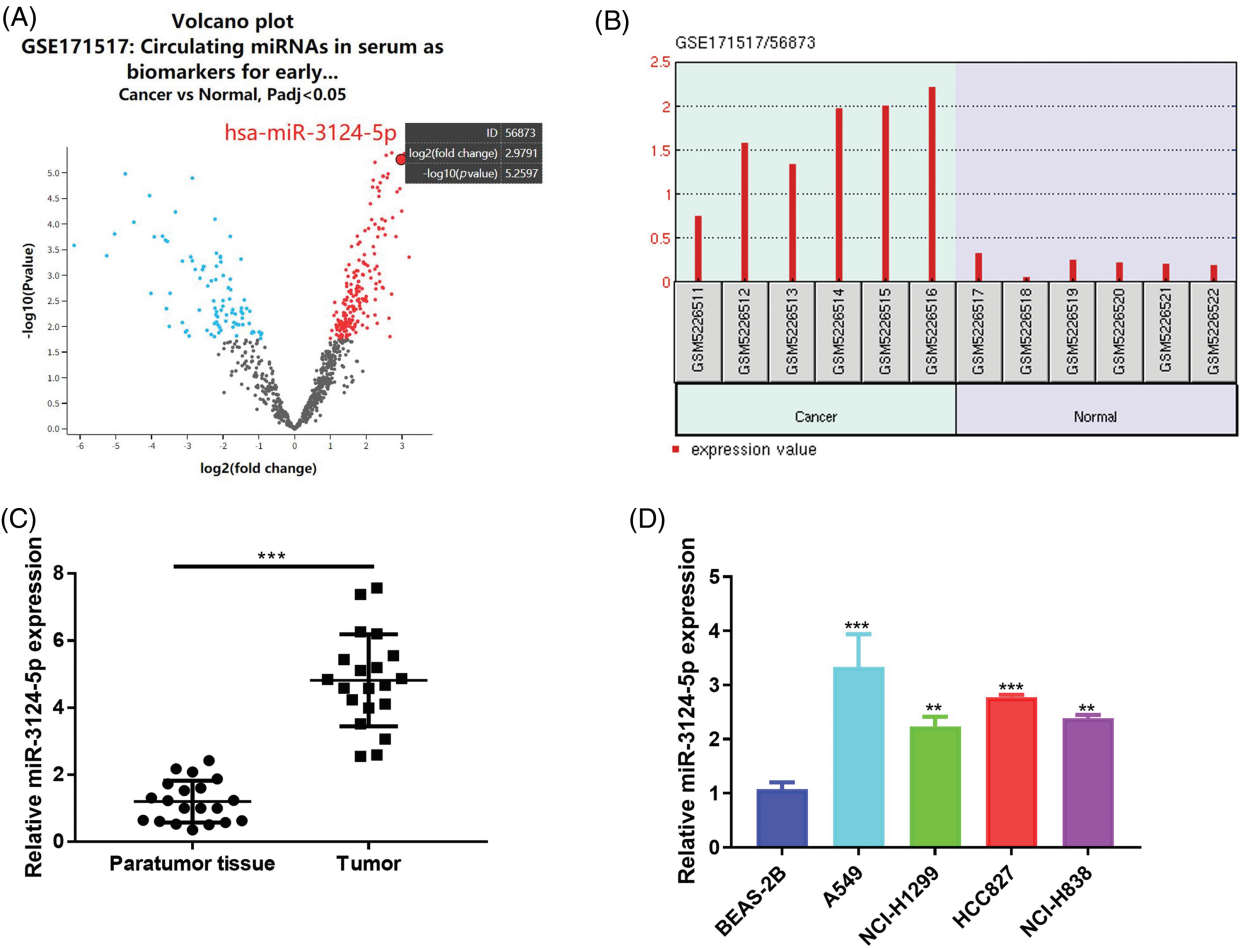
MiR-3124-5p was highly expressed in NSCLC. (A and B) GEO analysis (GSE171517) of differentially expressed miRNAs in serum between NSCLC patients and normal controls. (C) RT-qPCR was used to analyse the miR-3124-5p expression in NSCLC patient tissues (n = 20). (D) The expression of miR-3124-5p in NSCLC (A549, NCI-H1299, HCC827 and NCI-H838) and BEAS-2B cells. **p < 0.01; ***p < 0.001.

**Figure A1:**
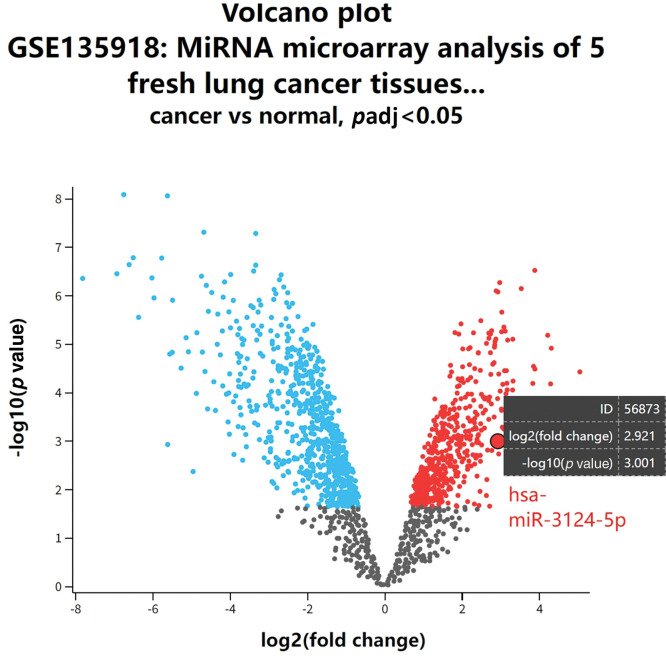
GEO database analysis (GSE135918) of differentially expressed miRNAs in cancer and non-cancer lung tissues of lung adenocarcinoma patients.

We conducted a bioinformatics analysis of the GSE171517 dataset using the GEO2R platform to screen for miRNAs with significant differences between the serum of NSCLC patients and normal subjects. As seen in Fig. 1A,B, miR-3124-5p expression was markedly greater in the cancer group than in the normal group (log_2_ (fold change) = 2.9791). The analysis of the GSE135918 expression profile also indicated that miR-3124-5p expression was markedly raised in LUAD (log_2_ (fold change) = 2.921) (Fig. A1). The miR-3124-5p expression levels in 20 pairs of NSCLC and corresponding paracancerous tissues were examined. The RT-qPCR experimental data demonstrated that miR-3124-5p expression was raised 2.8-fold in the tumour tissues when compared with the adjacent tissues (Fig. 1C). MiR-3124-5p expression was also prominently raised in NSCLC cell lines (A549 (2-fold), NCI-H1299 (1-fold), HCC827 (1.5-fold) and NCI-H838 (1.2-fold)) compared to BEAS-2B cells. A549 and HCC827 cells were the most significantly different (Fig. 1D). These data suggested that miR-3124-5p was highly expressed in NSCLC cells.

### CAF-exosomes transport miR-3124-5p to NSCLC cells

**Figure A2:**
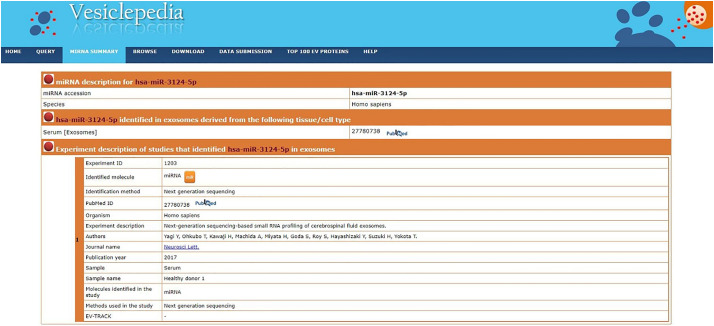
The Vesiclepedia database was used to analyze whether miR-3124-5p was present in exosomes.

Previous reports have found that the abnormal expression of miRNAs in serum is largely due to cell-to-cell interactions in the TME [33,34]. We first determined whether miR-3124-5p was present in exosomes using the online site Vesiclepedia. The results showed that serum exosomes contained miR-3124-5p (Fig. A2). Therefore, we hypothesised that miR-3124-5p in the TME plays a role in the cell-to-cell signalling processes of NSCLC.

**Figure 2:**
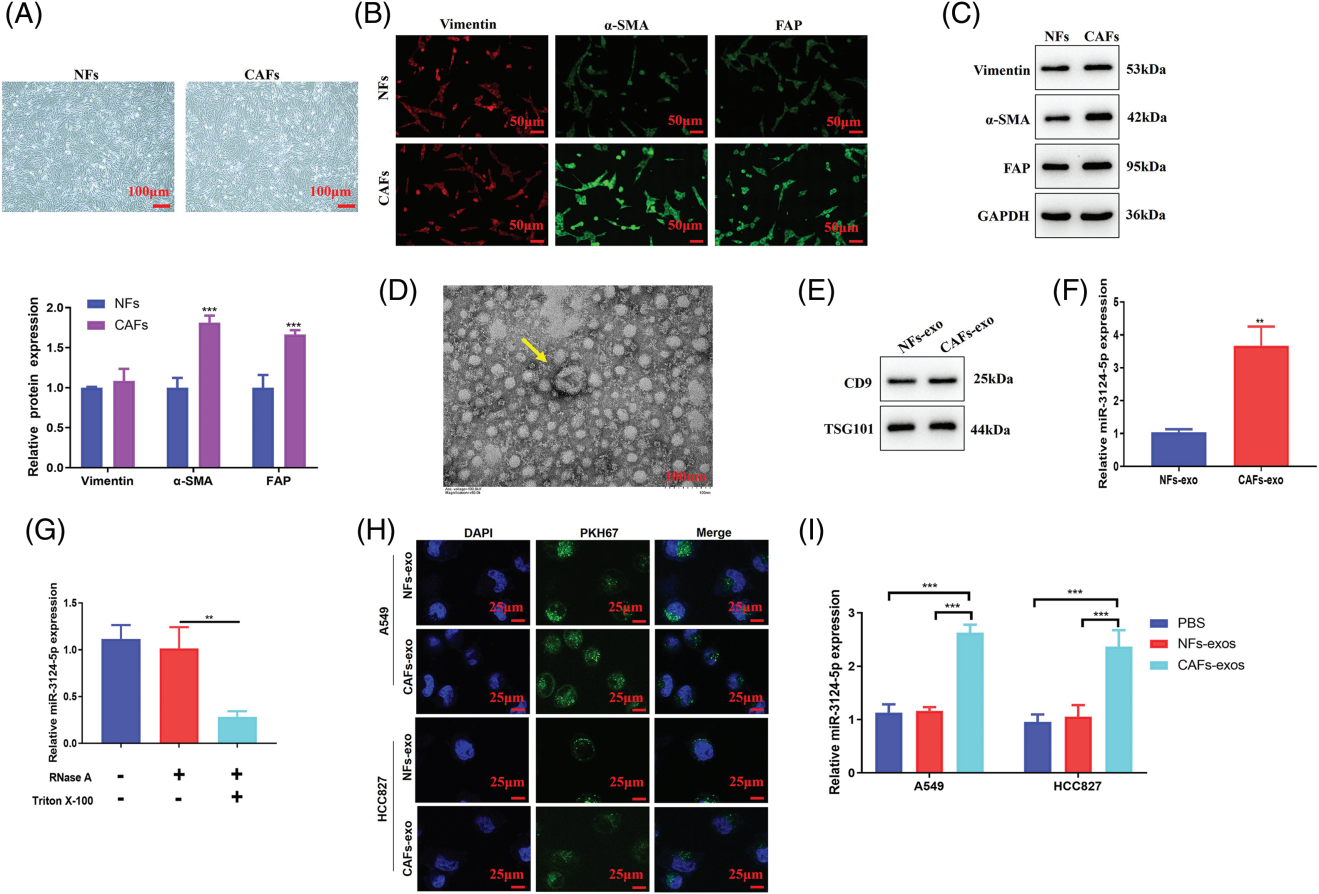
CAF-mediated exosomes delivered miR-3124-5p to NSCLC cells. (A) The shapes of NFs and CAFs were examined using a light microscope. (B and C) The vimentin, α-SMA, and FAP protein levels were measured via IF (B) and Western blot (C) analysis. (D) The feature and size (30–150 nm) of the exosomes were observed through TEM. (E) The CD9 and TSG-101 protein levels in exosomes extracted from NFs and CAFs were detected. (F) The miR-3124-5p expression in exosomes from the NFs and CAFs was tested. Blue represents NFs-derived exosomes group, and red represents the CAFs-derived exosomes group. (G) The expression of miR-3124-5p in CAFs was analysed using RT-qPCR after adding 2 mg/mL RNase treatment alone or in combination with 0.1% Triton X-100. Blue represents the untreated group (control), red represents the RNase A treatment group, and green represents the RNase A + Triton X-100 combined treatment group. (H) Visualisation of exosomes by the PKH67 probe. (I) NF- or CAF-derived exosomes were incubated with A549 or HCC827 cells, and the miR-3124-5p expression level in NSCLC cells was tested. **p < 0.01; ***p < 0.001.

CAFs play important roles in the TME [35]. Extensive data have found that CAFs participate in the regulation of multiple malignant tumour processes via the delivery of miRNAs [36,37]. Therefore, we isolated and identified NFs and CAFs. The shapes of NFs and CAFs are spindle-shaped (Fig. 2A). As seen in Fig. 2B, the IF data suggested that α-SMA and FAP in CAFs were markedly greater than those in NFs, but Vimentin was not significantly different. Consistently, the Western blot results suggested that α-SMA (increased 0.8-fold) and FAP (increased 0.7-fold) expression levels in CAFs were markedly greater than those in NFs, but the expression of Vimentin was not significantly different (Fig. 2C). These results indicated the successful isolation of CAFs. We cultured these cells and isolated and identified the exosomes. The ultrastructure of the CAF-derived exosomes was examined using TEM, and biomarkers (CD9 and TSG101) were determined via Western blot. As seen in Fig. 2D, E, exosomes were successfully obtained from these cells. To verify whether miR-3124-5p was overexpressed in the CAFs-exosomes, we measured miR-3124-5p expression in the NFs-and CAFs-exosomes. The miR-3124-5p expression was upregulated 2.5-fold in the CAF-exosomes when compared with the NF-exosomes (Fig. 2F). To demonstrate that miR-3124-5p was released via exosomes rather than via direct delivery, we treated CAFs with RNase and Triton X-100 separately or simultaneously and observed the changes in miR-3124-5p expression. RNase treatment did not change miR-3124-5p expression in CAFs, but cotreatment with RNase and Triton X-100 significantly reduced miR-3124-5p expression in CAFs by 70% (Fig. 2G). These results showed that miR-3124-5p was present in CAFs-secreted exosomes. We treated NSCLC cells with NF- or CAF-exosomes labeled with the PKH67 probe and found that these exosomes entered NSCLC cells (Fig. 2H). To further verify whether CAF-mediated exosomes delivered miR-3124-5p into NSCLC cells and promoted miR-3124-5p expression, we detected the miR-3124-5p expression level in NSCLC cells after co-incubation. As seen in Fig. 2I, the expression level of miR-3124-5p in the CAF exosome group was substantially increased by 1.2-fold (A549) and 1.5-fold (HCC827) compared to that in the PBS-treated group. However, there was no change in the expression level of miR-3124-5p in the NF exosome group.

### CAF-exosomes facilitated the malignant cell phenotypes of NSCLC through miR-3124-5p delivery

**Figure 3:**
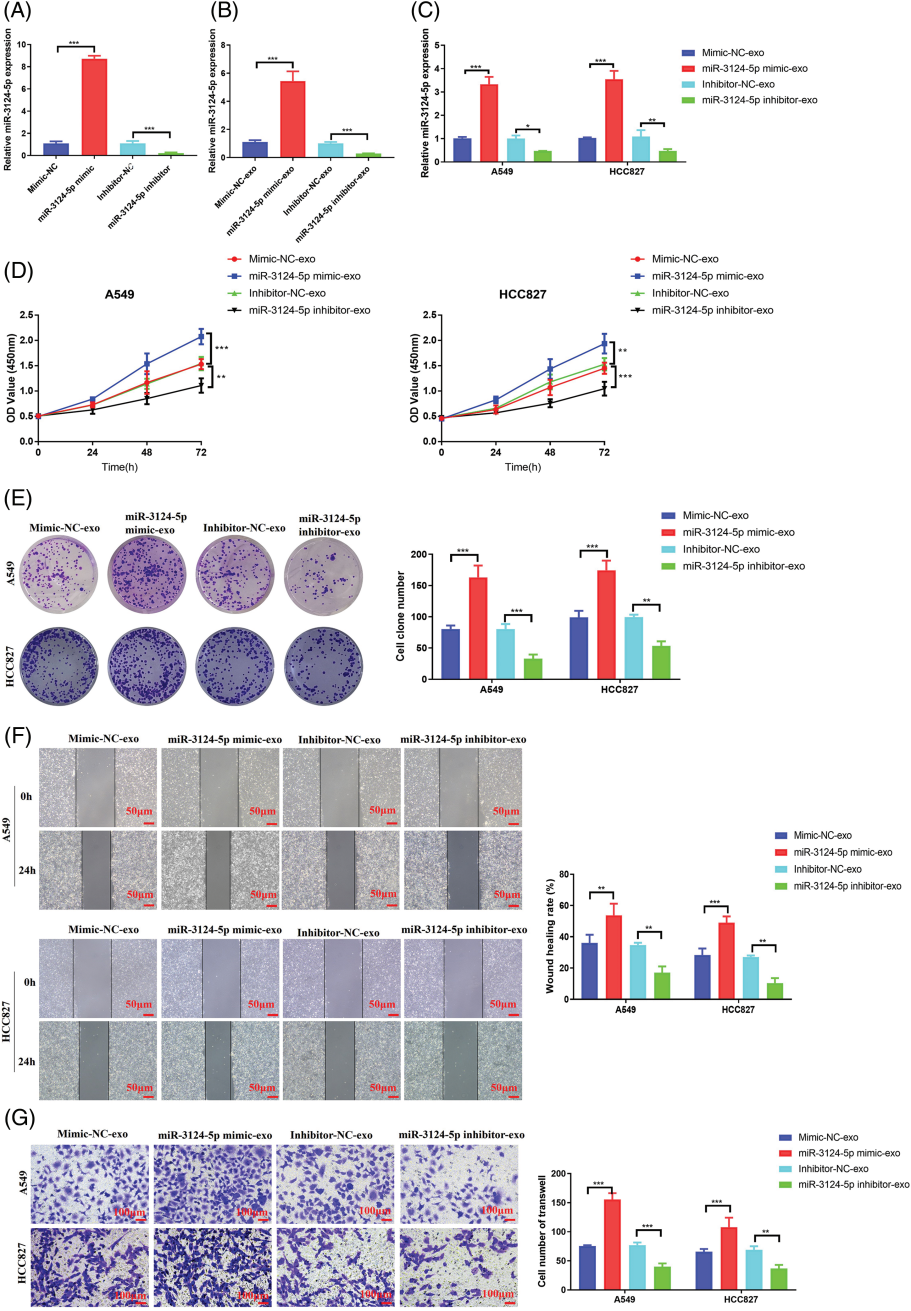
CAF exosomes facilitated the malignant cell phenotypes of NSCLC cells by delivering miR-3124-5p. (A) Detection of the transfection efficiency of miR-3124-5p-overexpressing/knockdown CAFs. (B) Detection of miR-3124-5p expression in exosomes derived from CAFs overexpressing/knocking down miR-3124-5p. (C–G) A549 and HCC827 cells were incubated with exosomes derived from CAFs overexpressing/knocking down miR-3124-5p: (C) the expression levels of miR-3124-5p, (D) CCK-8, (E) colony formation, (F) wound healing, and (G) Transwell experiments. *p < 0.05; **p < 0.01; ***p < 0.001.

Then, we explored the role of miR-3124-5p in CAFs-exosomes in NSCLC cells. As shown in Fig. 3A, we first overexpressed and knocked down miR-3124-5p in CAFs. The expression level of miR-3124-5p in the overexpression group increased 6.8-fold compared to that in the mimic NC group, and the miR-3124-5p expression in the knockdown group reduced by 90% in contrast with that in the inhibitor NC group. We extracted CAF-derived exosomes after transfection and examined the miR-3124-5p expression (Fig. 3B). The expression level of exosome-secreted miR-3124-5p in the mimic group was 3.8-fold greater than that in the mimic NC exosome group, and the expression level of exosome-derived miR-3124-5p in the knockdown group was 70% lower than that in the inhibitor NC exosome group. We treated NSCLC cells with the transfected CAF-exosomes and observed the changes in miR-3124-5p expression in the NSCLC cells. The miR-3124-5p-overexpressing group exhibited a 2.3-fold increase in the miR-3124-5p expression of A549 cells and a 2.4-fold increase in the HCC827 cells compared to the mimic NC exosome treatment group. The miR-3124-5p-knockdown group exhibited a 50% decrease in the miR-3124-5p expression of A549 cells and a 60% decrease in the miR-3124-5p expression of HCC827 cells compared to the inhibitor NC exosome treatment group (Fig. 3C). Subsequently, we observed changes in the cellular phenotypes of NSCLC after coincubation (treated as described above). The addition of CAF exosomes overexpressing miR-3124-5p significantly increased the proliferation of NSCLC cells (CCK-8: increased 0.4-fold (A549) and 0.3-fold (HCC827); colony formation: increased 1-fold (A549) and 0.8-fold (HCC827)). However, the proliferation of NSCLC cells was memorably decreased after coincubation with CAF exosomes in which miR-3124-5p was knocked down (CCK-8: decreased by 30% (A549) and 30% (HCC827); colony formation: decreased by 60% (A549) and 50% (HCC827)) (Fig. 3D, E). The wound healing assay demonstrated that the addition of CAF exosomes overexpressing miR-3124-5p significantly increased the migration ability of NSCLC cells by 1.1-fold (A549) and 0.6-fold (HCC827). In contrast, the migration ability decreased markedly after co-incubation with CAF exosomes in which miR-3124-5p was knocked down (decreased by 50% (A549) and 50% (HCC827)) (Fig. 3F). Transwell experiments revealed that the addition of CAF-exosomes over-expressing miR-3124-5p dramatically increased the invasion ability of NSCLC cells by 1.1-fold (A549) and 0.7-fold (HCC827). In contrast, the invasion ability was markedly reduced after coincubation with CAF exosomes in which miR-3124-5p was knocked down (decreased by 50% (A549) and 60% (HCC827)) (Fig. 3G).

### MiR-3124-5p sponges TOLLIP

**Figure 4:**
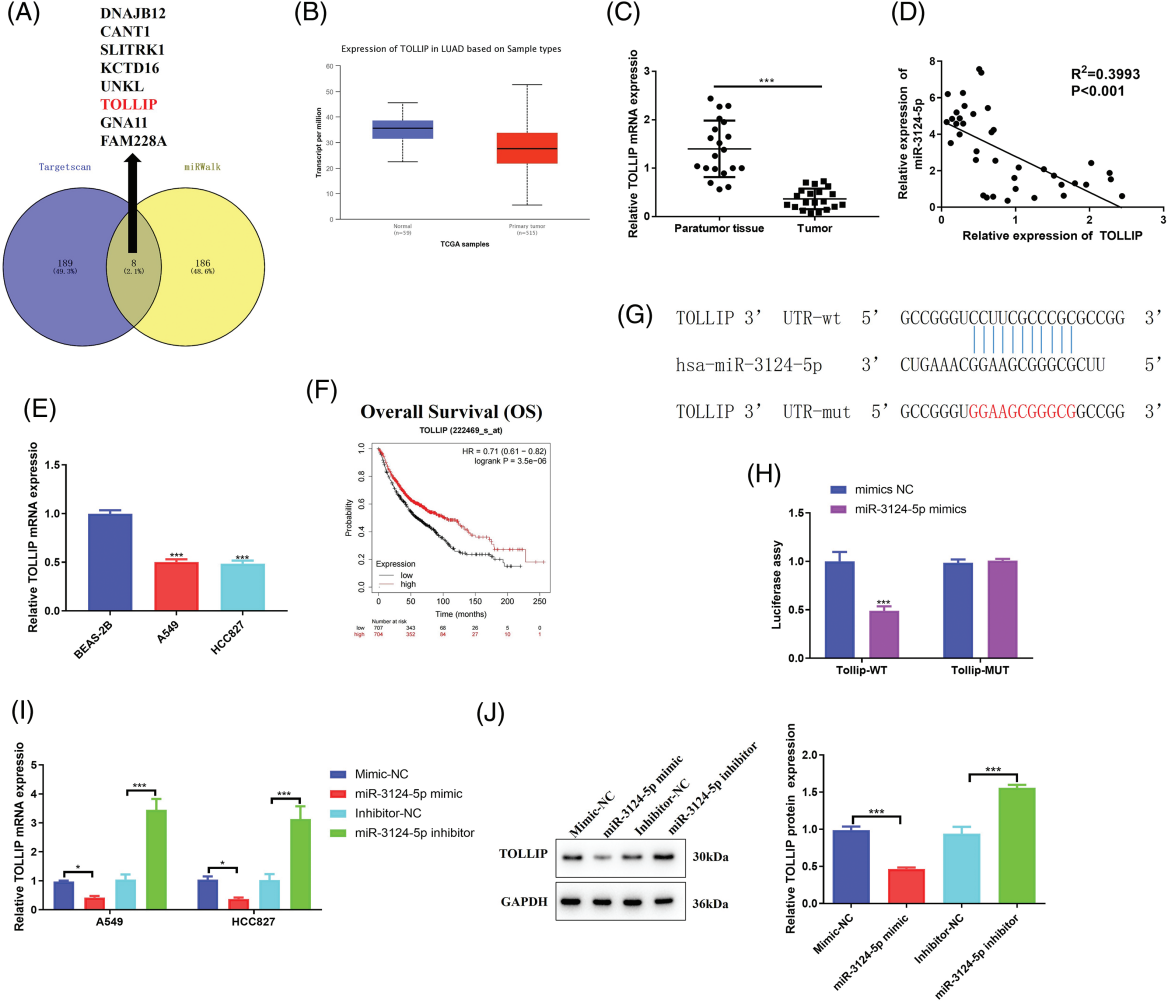
MiR-3124-5p sponges TOLLIP. (A) Venn diagram of the TargetScanHuman 7.2 and miRWalk (|energy| ≥ 25) websites applied to analyse the targets of miR-3124-5p. (B) The TOLLIP expression level in LUAD was predicted using the UALCAN online website (TCGA module). (C) The TOLLIP expression in NSCLC patient tissues was analysed using RT-qPCR (n = 20). (D) Pearson correlation analysis of miR-3124-5p and TOLLIP expression levels in NSCLC clinical samples. (E) The expression of TOLLIP in NSCLC (A549, NCI-H1299, HCC827, and NCI-H838) and BEAS-2B cells. (F) Correlation between TOLLIP expression and the prognosis of NSCLC patients. (G) The TargetScanHuman 7.2 database was applied to forecast the binding sequence of miR-3124-5p to TOLLIP. (H) Luciferase reporter assay. (I and J) After overexpression/knockdown of miR-3124-5p, the mRNA and protein expression levels of TOLLIP in A549 and HCC827 cells were detected by (I) RT-qPCR and (J) Western blot assays, separately. *p < 0.05; ***p < 0.001.

To further investigate the regulatory pathway of miR-3124-5p in NSCLC, we forecasted the prospective downstream targets regulated by miR-3124-5p using the TargetScan and miRWalk databases and identified 8 genes (Fig. 4A). A literature review [38] and bioinformatics analysis (Fig. 4B) identified TOLLIP as a downstream target gene of miR-3124-5p. We detected TOLLIP mRNA expression in NSCLC tissue samples and related cells. As seen in Fig. 4C, TOLLIP expression in NSCLC tumour tissues was markedly lower than that in adjacent tissues (a decrease of 70%). Pearson analysis between miR-3124-5p and TOLLIP in the clinical tissues of NSCLC patients revealed that miR-3124-5p expression was negatively related to TOLLIP (Fig. 4D). Consistent with this finding, the expression of TOLLIP was downregulated by 50% in A549 and HCC827 cells compared to BEAS-2B cells (Fig. 4E). We predicted the correlation between OS and TOLLIP expression via the Kaplan-Meier Plotter website. The data showed that the prognosis of NSCLC patients was positively correlated with TOLLIP expression (Fig. 4F). Luciferase experiments demonstrated that miR-3124-5p sponged TOLLIP (Fig. 4G, H). To further clarify the negative regulation of TOLLIP by miR-3124-5p, we individually overexpressed or knocked down miR-3124-5p in A549 and HCC827 cells. The data suggested that upregulating miR-3124-5p strongly inhibited TOLLIP expression (decreased by 50% (A549) and 70% (HCC827)), and knockdown of miR-3124-5p strongly stimulated TOLLIP expression (increased 2.3-fold (A549) and 2-fold (HCC827)) (Fig. 4I). Finally, we detected TOLLIP protein expression using Western Blot. The trend of protein results was consistent with the trend of mRNA level changes (Fig. 4J). These data indicated that miR-3124-5p directly negatively regulated TOLLIP expression.

### TOLLIP overexpression suppressed the malignant phenotypes of NSCLC cells via negative regulation of the TLR4/Myd88/NF-κB pathway

**Figure 5:**
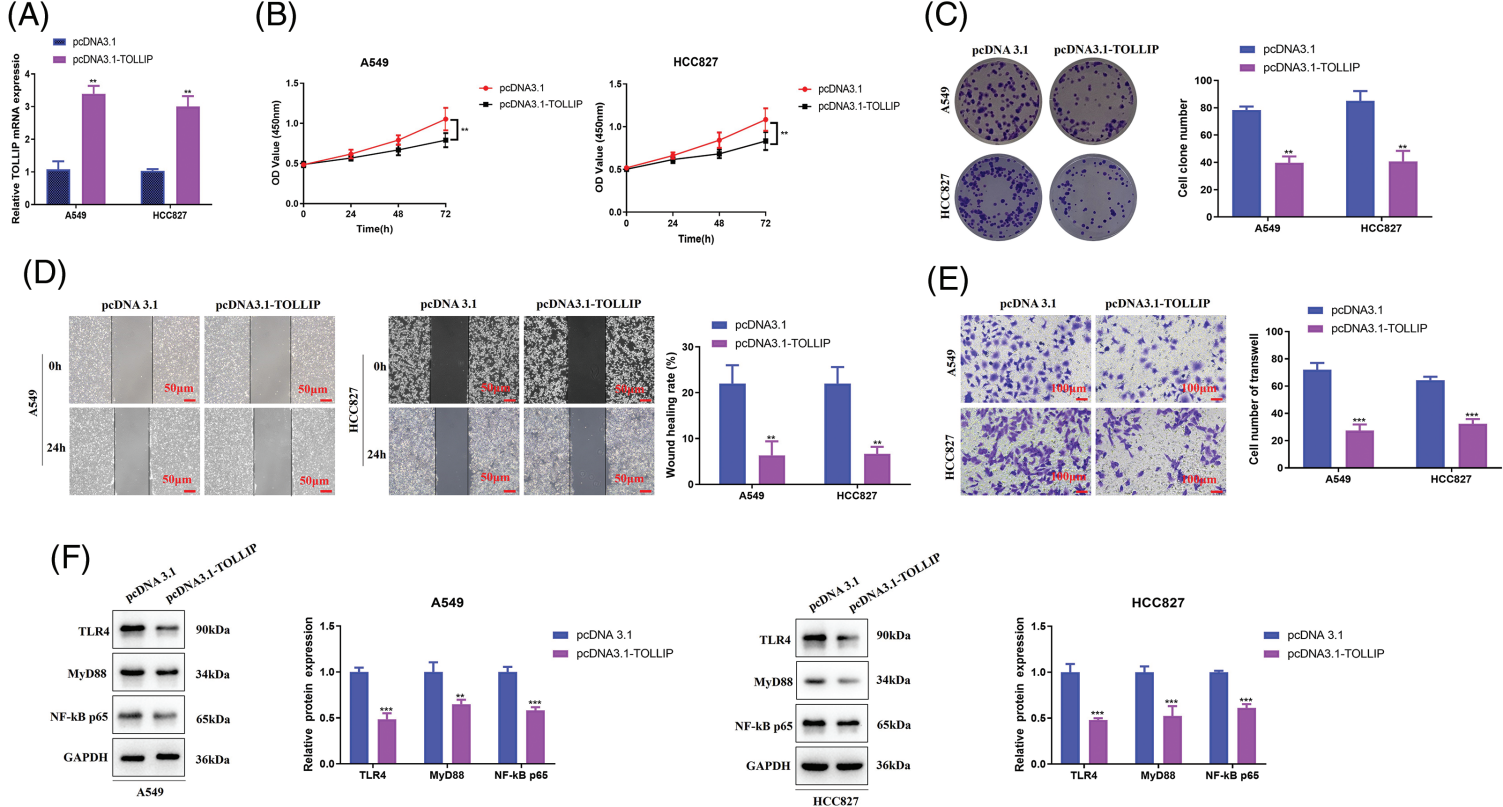
TOLLIP overexpression suppressed the malignant phenotypes of NSCLC cells via negative regulation of the TLR4/Myd88/NF-κB pathway. TOLLIP was overexpressed in A549 and HCC827 cells: (A) its overexpression efficiency by RT-qPCR, (B) CCK-8, (C) colony formation, (D) wound healing, (E) Transwell assays, and (F) Western blot assays to determine the TLR4, Myd88, and NF-κB p65 protein levels. **p < 0.01; ***p < 0.001.

TOLLIP is involved in the malignant process of cancers, and it is closely connected with the bad prognosis of cancer patients [39,40]. However, TOLLIP is poorly expressed in NSCLC tissues, and patients with high TOLLIP expression have a better prognosis [38]. These studies suggest that TOLLP functions differently in different tumours, and that its function in NSCLC has not been fully elucidated. Therefore, to further explore the regulatory function of TOLLIP in NSCLC, we transfected TOLLIP overexpression plasmids into A549 and HCC827 cells and successfully overexpressed TOLLIP (increased 2.1-fold (A549) and 1.9-fold (HCC827)) (Fig. 5A). We observed changes in the malignant phenotypes of the cells. Cell viability assays indicated that TOLLIP overexpression strongly suppressed the proliferation of NSCLC cells (CCK-8: decreased by 20% (A549 and HCC827); colony formation: decreased by 50% (A549 and HCC827)) (Fig. 5B, C). Wound healing experiments suggested that TOLLIP overexpression markedly suppressed the migration ability of NSCLC cells (decreased by 70% (A549 and HCC827)) (Fig. 5D). Transwell experiments showed that TOLLIP overexpression substantially inhibited the invasion ability of A549 and HCC827 cells (decreased by 60% (A549) and 50% (HCC827)) (Fig. 5E). A previous report revealed that TOLLIP negatively regulates TLR4 signalling [41]. The TLR4/Myd88/NF-κB signalling axis promotes the development of tumours [42]. Therefore, we tested the expression levels of TLR4 signalling pathway-related proteins. As seen in Fig. 5F, the protein levels of TLR4 (decreased by 50% (A549 and HCC827)), MYD88 (decreased by 40% (A549) and 50% (HCC827)), and NF-κB p65 (decreased by 40% (A549 and HCC827)) were memorably lower in the TOLLIP overexpression group than in the NC group.

### Upregulation of TOLLIP expression reduced the role of overexpression of miR-3124-5p in promoting the cellular progression of NSCLC

**Figure 6:**
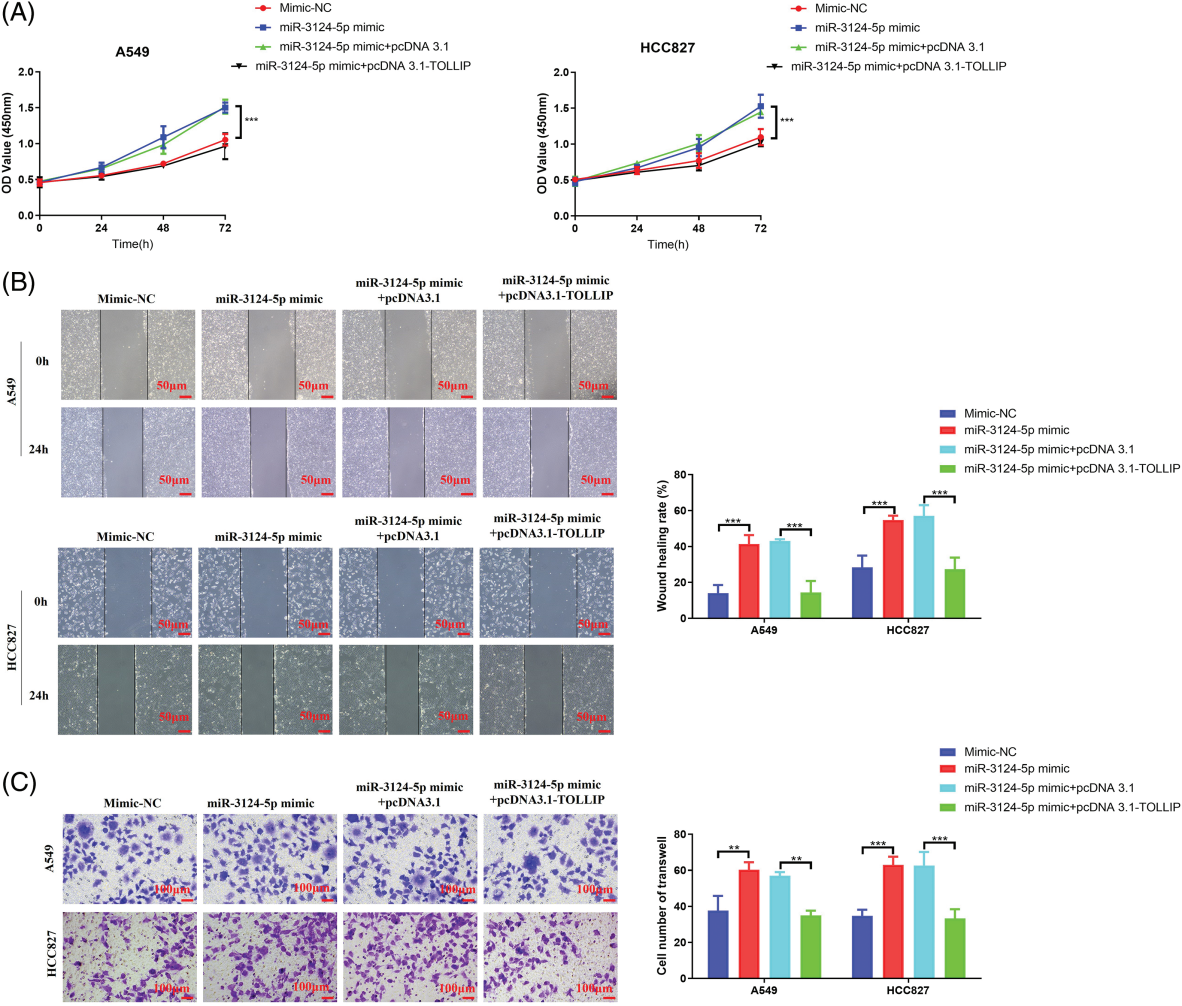
Upregulation of TOLLIP expression reduced the role of overexpression of miR-3124-5p in promoting the progression of NSCLC. TOLLIP pcDNA3.1 overexpression plasmid and miR-3124-5p mimic were simultaneously transfected into A549 and HCC827 cells to simultaneously overexpress TOLLIP and miR-3124-5p, and the changes of cell phenotype were observed. (A) CCK-8. (B) Wound healing. (C) Transwell assays. **p < 0.01; ***p < 0.001.

To further validate the miR-3124-5p-TOLLIP pathway in the malignant biological process of NSCLC cells, we conducted function rescue tests. TOLLIP pcDNA3.1 overexpression plasmid and miR-3124-5p mimic were simultaneously transfected into A549 and HCC827 cells to simultaneously overexpress TOLLIP and miR-3124-5p, and the changes of cell phenotype were observed. CCK-8 experiment indicated that upregulating miR-3124-5p significantly improved the proliferation of NSCLC cells (increased 0.4-fold (A549 and HCC827)), and the overexpression of TOLLIP reversed these effects (Fig. 6A). Scratch wound healing experiments suggested that overexpression of miR-3124-5p strongly stimulated migration (increased 2-fold (A549) and 0.9-fold (HCC827)), and overexpression of TOLLIP reversed this effect (Fig. 6B). Transwell assays also indicated that overexpression of miR-3124-5p strongly enhanced invasion (increased 0.6-fold (A549) and 0.8-fold (HCC827)), and overexpression of TOLLIP reversed this effect (Fig. 6C).

### Upregulation of TOLLIP expression weakened the promoting effect of exosomes derived from CAFs on NSCLC

**Figure 7:**
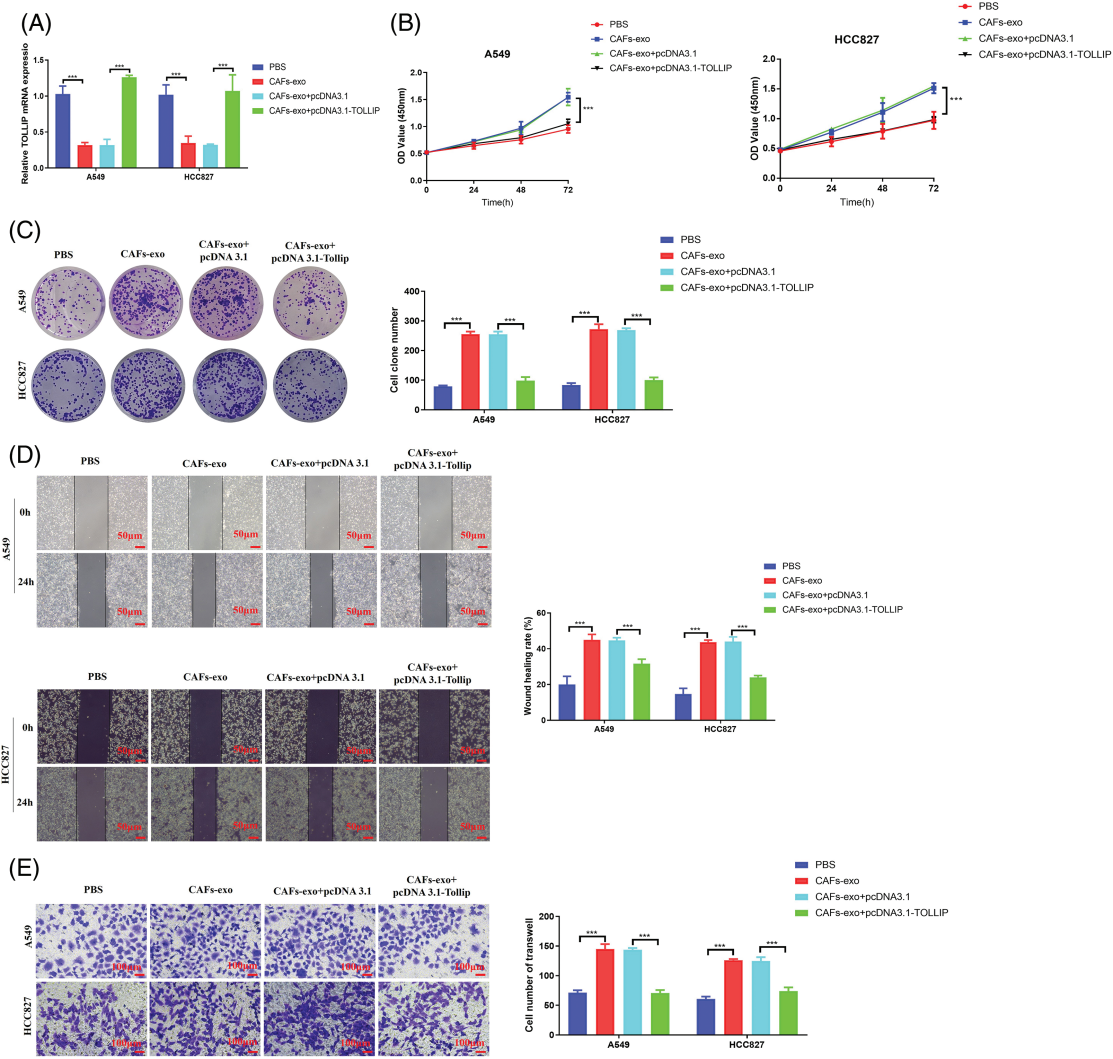
Upregulation of TOLLIP expression reduced the effect of exosomes secreted from CAFs on promoting NSCLC progression. (A) The expression levels TOLLIP were detected using RT-qPCR after coincubation with CAF-derived exosomes or transfection with the TOLLIP pcDNA3.1 overexpression plasmid. (B) CCK-8. (C) Colony formation. (D) Wound healing. (E) Transwell assays. ***p < 0.001.

To determine whether CAF-exosomes stimulated the biological process of NSCLC cells by suppressing the TOLLIP expression of NSCLC cells, we conducted rescue tests. We coincubated NSCLC cells that were not transfected or transfected with TOLLIP-overexpressing plasmids with CAF-derived exosomes and measured TOLLIP expression. As seen in Fig. 7A, the expression level of TOLLIP in the CAF exosome-treated group was decreased by 70% when compared with that in the PBS-treated group. However, TOLLIP expression in the CAF-exosome treatment + TOLLIP overexpression group increased 3-fold (A549) and 2.3-fold (HCC827) compared to that in the CAF-exosome treatment group. We examined changes in the malignant phenotype of the tumour cells. Cell viability tests indicated that CAF exosomes prominently increased the proliferation ability of NSCLC cells (CCK-8: increased 0.6-fold (A549 and HCC827), colony formation: increased 2.2-fold (A549) and 2.3-fold (HCC827)), and the overexpression of TOLLIP reversed these effects (Fig. 7B,C). Wound healing experiments suggested that CAF-derived exosomes strongly stimulated migration (increased 1.3-fold (A549) and 2-fold (HCC827)), and overexpression of TOLLIP reversed this effect (Fig. 7D). Transwell assays also indicated that CAF-derived exosomes strongly enhanced invasion (increased 1-fold (A549) and 1.1-fold (HCC827)), and overexpression of TOLLIP reversed this effect (Fig. 7E).

## Discussion

In the present study, we focused on investigating the function of miR-3124-5p secreted by CAFs in promoting the malignant biological phenotype of NSCLC cells. Our research enriches the evidence that exosomes derived from CAFs promote proliferation, migration, and invasion of NSCLC cells. Mechanistically, we found that miR-3124-5p secreted by CAFs exerts a pro-cancer effect by inhibiting TOLLIP expression and promoting the TLR4/MyD88/NF-κB signaling pathway. CAF-derived exosomes are important mediators of cancer development [29]. For example, CAF-derived exosomes promoted pancreatic cancer chemotherapy resistance by regulating the proliferation of cancer cells [43]. CAF-exosomes promoted cell migration and invasion of gastric cancer through the IL-32/ESR1 pathway [44]. The role of CAF-exosomes in NSCLC was recently reported [45]. However, the regulatory pathway has been less studied. Previous reports have found that CAF-derived exosomes are primarily involved in the regulation of NSCLC chemotherapy resistance, migration, and invasion processes via the delivery of miRNAs [7,28]. Therefore, the present study further examined the miRNAs contained in exosomes secreted from CAFs and the molecular pathways regulating the development of NSCLC. We determined that the serum miR-3124-5p was highly expressed in NSCLC patients. Exosomes deliver miR-3124-5p into cholangiocarcinoma cells to suppress GDF11 expression and promote the malignant progression of cholangiocarcinoma [46]. MiR-3124-5p may be a promising target for the incipient diagnosis and prognosis of small-cell carcinoma (SCLC) primarily because it may appear in the serum exosomes of SCLC patients [47]. We analysed the Vesiclepedia online database and identified the presence of miR-3124-5p in serum exosomes. All in all, these studies imply that miR-3124-5p released by exosomes plays a serious role in NSCLC, but the regulatory mechanisms involved have not been reported. For this reason, we hypothesised that miR-3124-5p was overexpressed in CAF-exosomes and participated in signalling crosstalk with tumour cells within the TME.

Therefore, we separated and identified CAF-derived exosomes from NSCLC clinical tissues. We illuminated that CAF-derived exosomes enriched in miR-3124-5p entered NSCLC cells. To elucidate the regulatory role of CAF-exosome-derived miR-3124-5p in NSCLC, we overexpressed/knocked down miR-3124-5p in CAFs and incubated these transfected cell-derived exosomes with NSCLC cells to monitor changes in cell phenotypes. The data indicated that CAF-exosomes accelerated the acquisition of malignant phenotypes in NSCLC cells via the delivery of miR-3124-5p. However, whether CAF exosomes promote the malignant phenotypes of NSCLC by delivering miR-3124-5p *in vivo* is not clear. In summary, we only validated our findings at the cellular level, which is one shortcoming of this study. Further construction of subcutaneous xenograft animal models is necessary for future research. MiR-3124-5p may be used as a serum marker for NSCLC patients only when it has been validated at the cellular and animal levels that CAF-mediated exosomes transport miR-3124-5p to promote the development of NSCLC.

We observed the expression levels of 8 target genes predicted by miR-3124-5p in NSCLC using the TCGA database. Among them, CANT1, SLITRK1, and UNKL expression levels were significantly overexpressed, while FAM228A (C2orf84) was downregulated, but not significantly. The expression levels of DNAJB12, KCTD16, TOLLIP, and GNA11 were significantly downregulated. Therefore, we conducted literature research on DNAJB12, KCTD16, TOLLIP, and GNA11. The research showed that TOLLIP was downregulated in NSCLC and closely linked to bad prognosis [38]. Therefore, TOLLIP was chosen as the downstream target for further research in this study. Bioinformatics analysis and RT-qPCR unveiled that the TOLLIP expression was low in NSCLC, and TOLLIP was positively associated with prognosis. Luciferase reporter and molecular biology experiments revealed that miR-3124-5p sponged and negatively adjusted the TOLLIP expression. However, the molecular mechanism of TOLLIP in NSCLC has not been reported. Therefore, we overexpressed TOLLIP in NSCLC cells and found that it suppressed the malignant progression of NSCLC cells. A previous report also revealed that TOLLIP negatively regulates TLR4 signalling [48]. In severe acute pancreatitis rats with acute lung injury, notoginsenoside R1 promotes TOLLIP expression by inhibiting miR-128-2-5p, thereby inhibiting the expression of TLR4, MyD88, and NF-κB related pathway proteins and exerting a protective effect [49]. In endotoxemia, PHLDA1 inhibits pro-inflammatory responses by repressing the TLR4/MyD88/NF-κB pathway through TOLLIP [41]. The TOLLIPTLR4/MyD88/NF-κB regulatory pathway has been mainly reported in inflammatory diseases, and it is still unclear whether it plays a regulatory role in cancer. The TLR4/Myd88/NF-κB axis promotes the development of tumours [42]. In this study, we examined TLR4/Myd88/NF-κB pathway in A549 and HCC827 cells after TOLLIP overexpression. The results were in line with expectations. In addition, we found in the literature that the expression levels of TLR4 [50]/MyD88 [51]/NF-κB [52] were significantly upregulated in clinical lung cancer samples. To validate that CAF-derived exosomes promoted malignant biological processes in NSCLC by regulating the expression of TOLLIP in NSCLC cells, we performed functional salvage experiments. The experimental data revealed that TOLLIP overexpression weakened the ability of CAF-derived exosomes to facilitate the malignant phenotype of NSCLC cells. Other reports have shown that TOLLIP is associated with cancer-promoting processes. For example, TOLLIP stimulates the proliferation, migration, and metastasis of hepatocellular carcinoma (HCC) cells [39]. TOLLIP also leads to insensitivity to chemotherapy in renal cell carcinoma by inducing autophagy [40]. These results suggest that the expression and function of TOLLIP in different tumours are heterogeneous and specific. These reports further highlight the necessity of studying the TOLLIP regulatory pathway in NSCLC. However, the contribution of the miR-3124-5p-TOLLIP regulatory axis to the CAF exosome-mediated promotion of NSCLC is not known. These questions include whether CAF-derived exosomal miR-3124-5p regulates genes other than TOLLIP to play a pro-cancer role and whether CAF-derived exosomes contain miRNAs other than miR-3124-5p to inhibit TOLLIP, which require further research. Our study only elucidated at the *in vitro* level that CAF-mediated exosome miR-3124-5p promotes the malignant progression of NSCLC cells by suppressing TOLLIP expression.

The limitation of this study is that it has not been further validated at the *in vivo* level. Our current results can only indicate that CAFs can cause signal crosstalk between secreted exosomes and NSCLC cells. However, this model cannot fully replicate the regulatory mechanisms *in vivo*. CAFs are crucial stromal cells in the TME, so *in vitro* studies may not accurately represent the true TME conditions. Therefore, it is necessary to establish a nude mouse subcutaneous tumour model to assess the impact of exosomes derived from CAFs on tumour formation. Through pathological examination, we can observe how CAFs influence the malignant progression of NSCLC as a whole.

## Conclusion

We illuminated that CAF-mediated exosomal miR-3124-5p sensitised NSCLC cells to the TLR4/Myd88/NF-κB axis by inhibiting the expression of TOLLIP to promote malignant biological processes in NSCLC. This study provides an interesting direction for the diagnosis and treatment of NSCLC.

## Data Availability

The datasets generated during and/or analyzed during the current study are available from the corresponding author on reasonable request.
